# Appendiceal Mucinous Tumor Presenting as Recurrent Bowel Obstruction

**DOI:** 10.3390/diagnostics12112832

**Published:** 2022-11-17

**Authors:** Wei-Tang Lin, Yen-Hsiang Wang, Wei-Yu Chen, Wilson T. Lao

**Affiliations:** 1 Department of Radiology, Wan Fang Hospital, Taipei Medical University, Taipei 116, Taiwan; 2 Department of Radiology, School of Medicine, College of Medicine, Taipei Medical University, Taipei 110, Taiwan; 3 Department of Surgery, Wan Fang Hospital, Taipei Medical University, Taipei 116, Taiwan; 4 Department of Surgery, School of Medicine, College of Medicine, Taipei Medical University, Taipei 110, Taiwan; 5 Department of Pathology, Wan Fang Hospital, Taipei Medical University, Taipei 116, Taiwan; 6 Department of Pathology, School of Medicine, College of Medicine, Taipei Medical University, Taipei 110, Taiwan

**Keywords:** appendix, bowel obstruction, appendiceal mucinous neoplasm, appendiceal tumor

## Abstract

Appendiceal mucinous tumors are rare, with variable malignant potential, and they are usually found incidentally. Clinical symptoms are nonspecific. Rarely, appendiceal mucinous neoplasm causes bowel obstruction and makes diagnosis more difficult. We present a case of an 84-year-old female who came to our emergency department having had abdominal fullness and constipation for 5 days. Ileus, due to an affected adhesion band, was diagnosed initially, and symptoms improved gradually under conservative treatment. However, 3 months later she presented to the emergency department again with abdominal pain and distension; small bowel obstruction due to adhesion was again diagnosed. Recurrent bowel obstruction prompted emergent surgery. Operative findings showed a whitish appendiceal tumor adhering to and directly invading the adjacent ileum, with a segment of herniated small bowel wedged in between, causing the obstruction. Upon reviewing the initial computed tomography scan, the dilated tubular structure of appendiceal tumor was misrecognized as small bowel loop; there was no surrounding inflammatory sign, leading to diagnosis difficulty. Instead of a common cause of bowel obstruction, such as adhesion band, this case revealed bowel obstruction can be caused by the direct invasion of an appendiceal tumor. Awareness of this condition with careful image evaluation of small bowel obstruction is essential for diagnosis.

**Figure 1:**
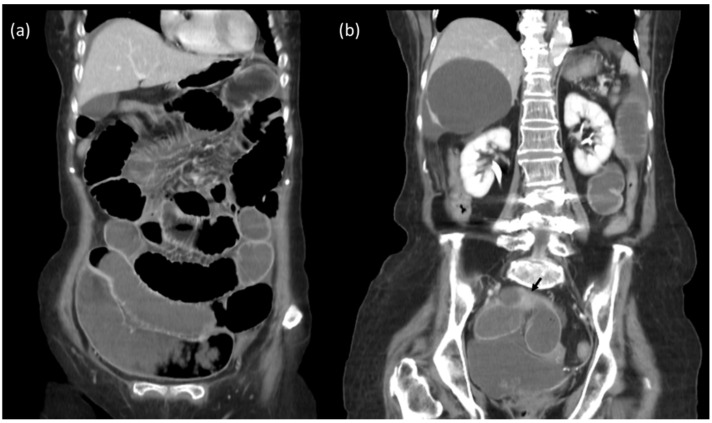
The 84-year-old female presented to emergency department having had abdominal fullness and constipation for 5 days. Initial KUB radiograph shows distended small bowels. Contrast-enhanced computed tomography of abdomen–pelvis: (**a**,**b**) Distended small bowel loops, with transition zone at ileum (black arrow). Mechanical obstruction due to adhesion was diagnosed.

**Figure 2:**
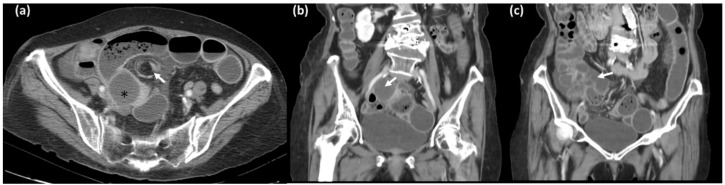
Three months later she presented to the emergency department again with abdominal pain and distension, they repeated the contrast-enhanced computed tomography of abdomen–pelvis: (**a**) Dilated small bowel loops, with whirl sign (arrow), which may indicate bowel volvulus or closed loop obstruction. A cystic structure adjacent to transition zone was found (*) suspected to be the enlarged appendix. (arrows in coronal image (**b**,**c**)) [1,2].

**Figure 3:**
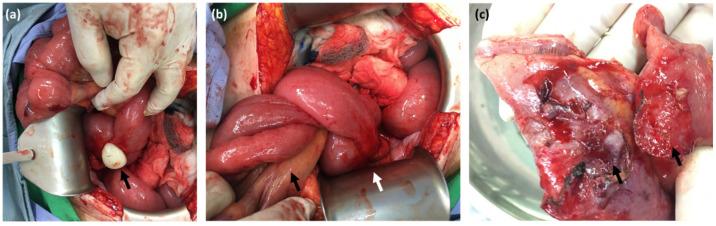
Operative findings: (**a**) Appendiceal mucocele (arrow). (**b**) Tumor caused small bowel adhesion (white arrow) and internal herniation (black arrow). (**c**) Tumor capsule adhered to serosa of ileum (arrows). Bowel obstruction caused by appendiceal tumor was rarely reported [3,4,5,6,7].

**Figure 4:**
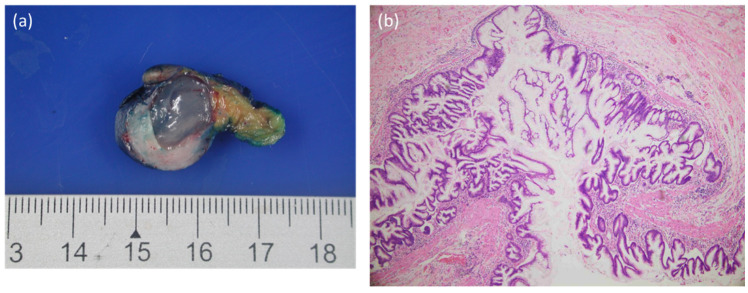
The microscopic examination of appendiceal tumor revealed low-grade appendiceal mucinous neoplasm, which was adherent to the adjacent small bowel. (**a**) Low-grade appendiceal neoplasm with a dilated lumen filled with mucus. (**b**) Low-grade mucinous appendiceal neoplasm lined by filiform mucinous epithelium. The mucinous epithelium displayed no obvious nuclear pleomorphism [8,9].

## Data Availability

All data are available within the article.
